# Serum vitamin D and body composition in adults undergoing fitness assessments: A correlation study

**DOI:** 10.1371/journal.pone.0197903

**Published:** 2018-06-01

**Authors:** LesLee Funderburk, Matthew Peterson, Nish Shah, Meredith Morgan, Peter Grandjean

**Affiliations:** 1 Robbins College of Health and Human Sciences, Baylor University, Waco, Texas, United States of America; 2 Orthopedics and Sports Medicine, Houston Methodist, Sugarland, Texas, United States of America; Charles P. Darby Children's Research Institute, 173 Ashley Avenue, Charleston, SC 29425, USA, UNITED STATES

## Abstract

The purpose of this descriptive study was to assess serum levels of 25(OH) vitamin D_3_ (25(OH)D) in participants who voluntarily participated in a fitness assessment and correlate this with muscular strength, weekly exercise, overall body composition, and dietary intake of vitamin D and calcium. Thirty-six participants were recruited. Anthropometric measurements and handgrip strength were taken with blood analyses completed utilizing 25-hydroxyvitamin D assay. A dietary screening survey was used to assess calcium and vitamin D intake. All data collection was completed at time of assessment appointment. Statistical analyses completed utilized Pearson’s and Kendall’s Tau correlation, with level of significance set at p < 0.05. Twenty-two percent of our sample were 25(OH)D deficient and 33% 25(OH)D insufficient, with 16% consuming adequate vitamin D and 5% consuming adequate calcium. Eight of the females and one male had below average DXA assessments for bone health. Vitamin D intake was significantly correlated with serum 25(OH)D levels (τ = 0.29, *p*<0.01). In females there was an inverse relationship between 25(OH)D and weight (*r* = -0.45, *p*<0.05). Thirty-one percent of participants had higher than desirable waist circumference and were 25(OH)D insufficient or deficient. This sample had a high rate of 25(OH)D insufficiency and deficiency, with most not consuming adequate amounts of calcium and vitamin D. Normalizing serum 25(OH)D through food and supplements has the potential to positively impact several parameters of an individual’s health including weight status, visceral adiposity and waist circumference, and bone health.

## Introduction

Vitamin D is a nutrient with important roles in cardiovascular and musculoskeletal function [1]. Up to 41% of US adults are classified as vitamin D deficient, defined by the Endocrine Society® as a serum 25-hydroxy vitamin D3 (25(OH)D) concentration less than 20 ng/mL [1, 2]. Durazo-Arvizu et al [3] found that risk of death from all causes increased with 25(OH)D levels below 40 ng/mL. Osteoporosis risk increases as 25(OH)D levels decrease [4]. Adequate serum 25(OH)D has been correlated with muscle strength and the ability to improve strength [5, 6]. Given the many important roles that 25(OH)D has in the human body it is essential for individuals to routinely have their 25(OH)D status assessed and seek treatment as necessary.

Vitamin D levels are inversely correlated with weight status in adults [7, 8]. Overweight individuals that lose weight, have a subsequent increase in serum 25(OH)D. Cheng et al [9] found an inverse association between serum 25(OH)D and waist circumference, a surrogate measure of visceral fat [10]. Visceral versus subcutaneous fat is considered more detrimental to overall health, as it increases the risk of a variety of diseases [11]. Appropriate dietary intake of vitamin D and adequate serum 25(OH)D status may play a role in weight loss and weight maintenance. Some studies suggest that levels above 30 ng/mL are optimal for overall health and may be required to support exercise regimens sufficient to maintain a healthy body composition [12, 13].

Adequate intakes of vitamin D through food, supplements, and environmental exposure can help support healthy body composition and in turn strength [12, 14, 15]. An additional nutrient of concern is the mineral calcium. Calcium works in conjunction with vitamin D to support bone health with adequate intakes of vitamin D promoting increased intestinal absorption of calcium. According to the 2015 Dietary Guidelines for Americans report, increasing intake of vitamin D fortified dairy foods is one of the recommended shifts to make concerning diet. Fortified dairy products are one of the few food choices that are considered a good source of vitamin D. The average daily intake of dairy by U.S. adults is well below recommendations with a downward trend in intake for men and women as age increases [14].

Adults located in the central Texas region have the opportunity to participate in a curriculum-based community service program offered in collaboration with a local family medicine residency program. The assessments offered include a blood biochemical analysis, body composition assessments, and a general evaluation of the individual’s strength and cardiovascular fitness. This information is then provided to the participants to help them improve physical fitness via an individualized exercise prescription. Nutrition education or screening is not currently offered to these clients. Offering nutrition education in the future can be an added benefit to these clients for overall health improvement, once over-arching nutrition education needs are established.

The purpose of this descriptive study was to assess 25(OH)D status in participants who voluntarily participated in the assessments and correlate this with muscular strength, weekly exercise, overall body composition, and dietary intake of vitamin D and calcium. It was hypothesized that those who were 25(OH)D deficient or insufficient would perform less than optimally on strength assessments, have lower weekly exercise quantity, be overweight or obese, have elevated waist circumference, elevated levels of visceral fat, below normal bone health and have inadequate intakes of both calcium and vitamin D.

## Materials and methods

Thirty-six participants were recruited at the university exercise laboratory where assessments are conducted. Male and female participants were eligible if they were taking part in the assessments. Demographic variables include age, sex, and race/ethnicity.

This was a cross-sectional descriptive study, approved by the Baylor University Institutional Review Board and written informed consent was obtained from all participants. Recruitment occurred between January and April 2017, with all data collection completed at time of assessment appointment.

### Strength and exercise assessment

Participant’s grip strength was evaluated using a hand dynamometer (Hydraulic Hand Dynamometer, Baseline® Evaluation Instruments) as part of the assessment. The individual squeezes the dynamometer with maximum isometric effort, which is maintained for approximately five seconds, with measurement output provided in pounds [16]. Routine exercise participation was assessed from self-reported number of exercise sessions, of any type, they engaged in per week. This was then multiplied by the number of minutes per session and used as an indicator of routine exercise quantity. Each participant completed the Bruce treadmill protocol, used to predict VO_2_max [16]. The amount of time elapsed during treadmill exercise following this protocol was used as an indirect marker of cardiorespiratory fitness.

### Blood biochemistry

The biochemical marker evaluated was serum 25(OH)D. All blood draws were completed using trained lab staff. The blood analyses were completed and reported by Clinical Pathology Laboratories (Waco, TX) utilizing a 25-hydroxyvitamin D assay that measured 25- hydroxyvitamin D2 and D3, both of which contribute to active serum 25(OH)D levels [1]. The methodology utilized was chemiluminescent immunoassay. The Endocrine Society® defines deficiency as below 20 ng/ml and insufficiency as 21–29 ng/ml [1].

### Dietary intake

In order to assess nutrition intake, participants were interviewed to obtain a 24-hour dietary recall, following the basic guidelines of the multiple-pass approach [17]. The recalls were then analyzed using the Automated Self-Administered 24-hour recall (ASA24-2016), a web-based tool developed by National Cancer Institute (NCI) researchers [18]. The web-based option specifically for research was utilized. The participants also completed a short dietary screening survey to assess routine calcium and vitamin D intake. The screening tool was developed and validated by researchers at Nutrition Quest from the NHANES 1999–2001 dietary recall data and includes 19 food items, three supplement questions, and questions to adjust for food fortification practices all to specifically focus on routine calcium and vitamin D intake [19].

### Anthropometrics and body composition

All data collection occurred in person. Height was measured to the nearest 0.1 cm using a stadiometer with participants in exercise clothes, without shoes, but in stocking feet. Weight was measured to the nearest 0.10 kilogram on a calibrated, digital scale (Tanita®, SC 331S). Standardized waist circumference was measured per the National Heart, Lung, and Blood Institute (NHLBI) guidelines [20], and body fat percentage, visceral fat and bone status were measured using dual energy x-ray absorptiometry (DXA) bone densitometer (Hologic® Discovery QDR, Bedford, MA). Low bone mass was defined as having a T-score of <-1 for whole body bone density. Body Mass Index (BMI) was calculated as weight (kilograms) divided by height (meters) squared. The Fat Mass Index (FMI) was calculated as fat mass in kilograms divided by height (meters) squared [21]. The FMI classification ranges by Kelly et al [22] were used to categorize the participants and range from severe fat deficit up to obese class III. See Table 1. Visceral fat evaluation and metabolic risk factor levels for coronary heart disease are reported and interpreted via the scientific paper written by Kelly [23].

**Table 1:** Demographic characteristics of participants.

Averages	Men n = 19	Women n = 17
Age (years)*	54±17	56±11
Height (cm)	180 ± 8	161 ± 6
Weight (kg)	93 ± 13	72 ± 12
Waist Circumference (cm)	100 ± 10	101 ± 14
Fat free mass (kg)	68 ± 8	45 ± 7
Fat mass (kg)	25 ± 8	29 ± 8
Below Average Bone Mass	1	8
Body Mass Index (kg/m2)		
Normal (18.5–24.9)	3	5
Overweight (25–29.9)	11	8
Obese (30–34.9)	4	4
Obese class II (35–39.9)	1	0
Fat Mass Index (kg/m2)		
Normal		
Men: 3–6	7	
Women: 5–9		5
Excess fat		
Men: >6 to 9	5	
Women: >9 to 13		7
Obese class I		
Men: >9 to 12	7	
Women: >13 to 17		5
Serum 25(OH)D levels		
Normal	9	5
Insufficient	6	6
Deficiency	5	3

*reported as mean ± standard deviation

Fat Mass Index (FMI) calculated as fat mass in kilograms divided by height (meters) squared. Body Mass Index (BMI) calculated as weight (kilograms) divided by height (meters) squared.

### Statistical analyses

Variables were first assessed for gender differences with an independent samples *t*-test for parametric data or an independent samples Komogorov-Smirnov test for non-parametric data. Variables with statistically significant gender differences were analyzed with separate gender categories. Pearson’s correlation was used for normally distributed variables and Kendall’s Tau correlation was used for non-normally distributed variables. The level of significance was set at p < 0.05. Missing data were removed using pairwise deletion. All analyses were conducted SPSS version 24.0.

## Results

### Participants

The sample consisted of 53% males and 47% females with a mean age of 54.6. Most of our subjects were non-Hispanic white (89%), and all were non-smokers. Five participants with overweight BMI were at normal fat levels by FMI; two classified as normal weight by BMI were considered at excess fat by FMI; and three classified as overweight BMI were considered obesity class 1 by FMI. Eight of the females and one male had below average DXA assessments for bone health (Table 1). Twenty-two percent of our sample were 25(OH)D deficient and 33% 25(OH)D insufficient, with only 16% of participants consuming adequate amounts of vitamin D. The second nutrient of interest, calcium, was lower at 5% of the sample with adequate consumption (Table 2).

**Table 2:** Intake of select macro and micro nutrients.

% kcal Carbohydrate	46±10
% kcal Sugar	8±6
Fiber, gm	17±8
% kcal fat	36±9
% kcal saturated fat	11±4
% kcal protein	19±4
Calcium, mg, food & supplements	532±374
Vitamin D, IU, food & supplements	694±1202

Since vitamin D intake was not normally distributed in our sample, a Kendall’s Tau correlation was run, finding that vitamin D intake was significantly correlated with serum 25(OH)D levels (τ = 0.29, *p*<0.01). Similarly, greater levels of calcium intake were also associated with greater 25(OH)D levels (τ = 0.21, *p*<0.05). In our study we found that age was positively correlated with vitamin D intake (τ = 0.40, *p*<0.001). A significant relationship was found between visceral adipose tissue mass and total fat weight (τ = 0.55, *p*<0.001). See Table 3. Regarding visceral fat diagnostic thresholds, 41% of participants with sub-optimal or insufficient 25(OH)D levels were also categorized as either increased risk or high risk for coronary heart disease [23]. See Table 4.

**Table 3:** Statistical results. Data supporting these results can be accessed via the link provided in Supporting information below.

Category	*P* value
Serum 25(OH)D & vitamin D intake	τ = 0.29, *p* < 0.01*
Serum 25(OH)D & calcium intake	τ = 0.21, *p* < 0.05
Visceral adipose tissue mass & total weight	τ = 0.55, *p* < 0.001
Age & vitamin D intake	τ = 0.40, *p* < 0.001
Females—Serum 25(OH)D & weight	*r* = -0.45, *p* < 0.05**
Females—Great grip strength & lower FMI value	*r* = -0.30, *p* = 0.35
Females—Increased treadmill time & weight	*r* = -0.07, *p* > 0.05
Males—Serum 25(OH)D & weight	*r* = -0.29, *p* > 0.05
Males—Great grip strength & lower FMI value	*r* = -0.58, *p* < 0.01
Males—Increased treadmill time & weight	*r* = -0.49, *p* < 0.05

*Kendall’s Tau,

**Pearson’s correlations

**Table 4:** Frequency table showing serum 25(OH)D level categories and visceral adipose tissue area category.

		VAT area category		
		Normal (<100cm^2^)	Increased Risk (100-160cm^2^)	High Risk (>160cm^2^)
VitD Category	Insufficient (<20ng/mL)	2	4	2
	Sub-optimal (20-29ng/mL)	4	5	3
	Optimal (≥30ng/mL)	4	5	5

Variables that were found to be significantly different by gender include: grip strength, total fat, weight, and FMI (*p*<0.05). In females there was an inverse relationship between 25(OH)D and weight (*r* = -0.45, *p*<0.05), indicating that larger weights were associated with lower levels of 25(OH)D. In males, a positive relationship was witnessed between 25(OH)D and weight; however, this correlation was not statistically significant (*r =* 0.29, *p*>0.05). For males, a greater grip strength was correlated with a lower FMI value (*r* = -0.58, *p*<0.01). A similar relationship was witnessed in females, but was not statistically significant (*r* = -0.30, *p =* 0.35). Increased time on the treadmill was negatively correlated with weight in males, (*r* = -0.49, *p*<0.05). In females, a similar, but non-statistically significant trend was observed (*r* = -0.07, *p*>0.05). See Table 3.

## Discussion

This study evaluated the prevalence of 25(OH)D deficiency and insufficiency in a sample of participants taking part in comprehensive health and fitness assessments. We found that 33% of participants were 25(OH)D insufficient and 22% deficient; lower than the national average [1]. In this mainly non-Hispanic White sample from central Texas, the level of deficiency may be lower due to routine exposure to sunlight in this location at moderate latitude. That said, since it is known that a variety of biological systems respond better when 25(OH)D levels are ≥30 ng/ml it would be prudent to advise those in the insufficient range to consume more vitamin D rich foods or supplements [3, 4, 12, 13]. In these participants vitamin D intake from food sources and supplements had a significant correlation to 25(OH)D (p = 0.009).

Nine participants, eight being women, had below average DXA assessments for bone health, with an age range of 49 to 64 years. This number is concerning as it accounted for 47% of the women in this study. Of these, seven had less than desirable intakes of vitamin D and calcium. Vitamin D is a prohormone and plays a key role in calcium balance and subsequent bone structure. When these nutrients are consumed consistently in adequate amounts, this can promote healthy bone structure [24]. Physical activity of all types is also known to have a positive impact on bone health [25]. In this sample, it was found that bone density category had a significant relationship to participants exercise quantity (p<0.001).

Unlike results from other studies, we did not find a significant association between grip strength, exercise quantity or time on treadmill and 25(OH)D [5, 6]. Higher 25(OH)D concentrations have been linked with both leg and arm muscle strength, after adjusting for age and gender. The25(OH)D deficiency affects type II skeletal muscle fibers and may produce myopathy. Men and women with adequate serum 25(OH)D have greater muscle strength, and serum levels have been shown to be associated with grip strength [6, 26, 27]. Interestingly when we divided the group into women and men, there was a significant (p˂0.01) association in grip strength and exercise quantity in women but not in men. In men we found an inverse association between time on treadmill and body weight and inverse association of grip strength and FMI; inferring that excess weight and body fat in the men may be curtailing physical fitness. Excess weight may make it difficult to participate in routine physical activity, which is known to decrease the risk of metabolic and cardiovascular diseases [28].

**Fig 1:**
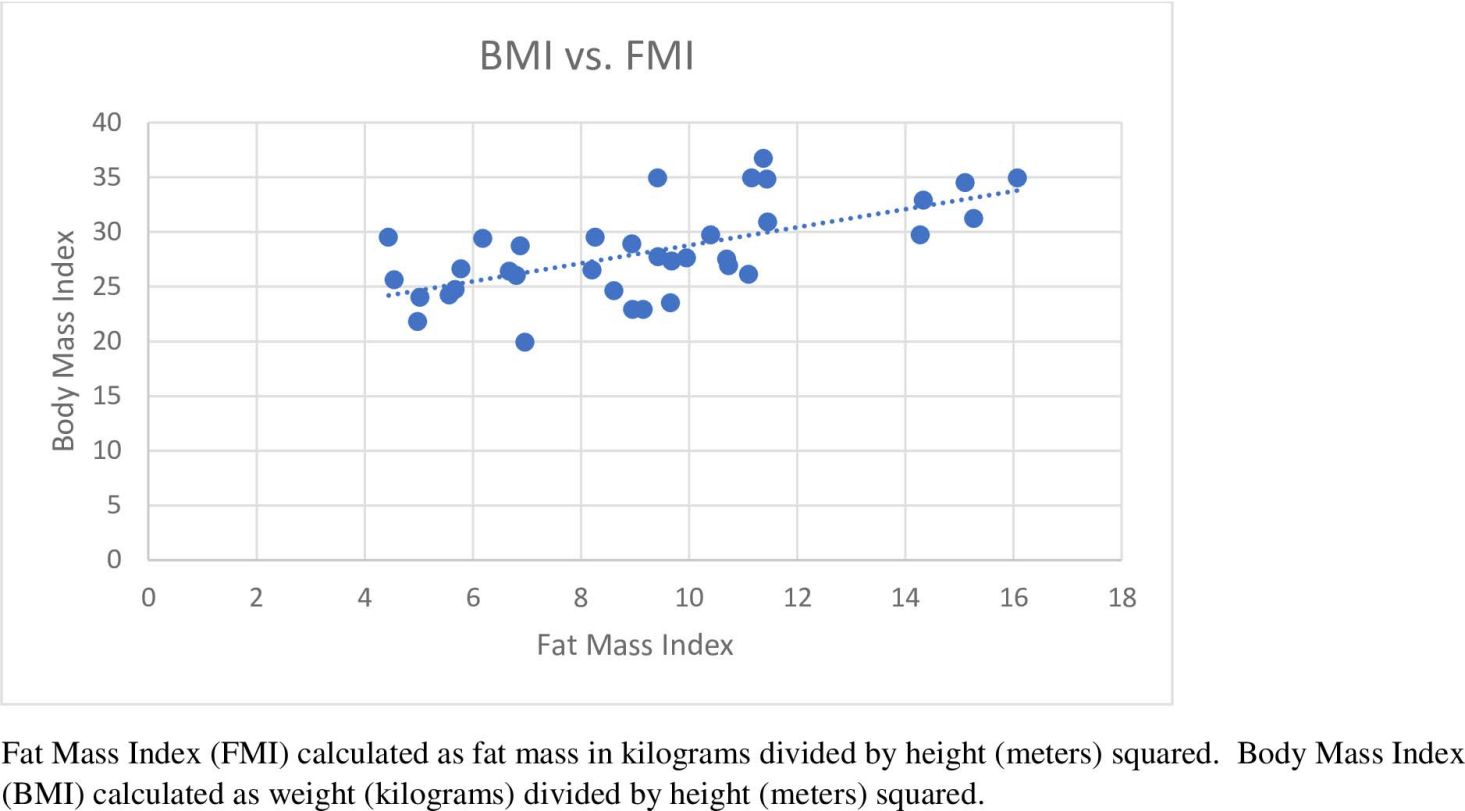
Correlation of BMI and FMI.

We categorized the participants by BMI and Fat Mass Index (FMI). VanItallie et al [21] first suggested this index in a nutrition assessment focused study, as it utilizes fat mass and height only, giving a truer indicator of adiposity. It was found that 72% of the participants matched similar weight status categories of BMI and FMI. This finding is further underscored by the strong, positive correlation between BMI and FMI in our cohort (*r* = 0.623, p<0.001), see Fig 1. This study is one of a few to use the FMI as a method for categorizing subject adiposity. There was a significant inverse association with 25(OH)D and weight status in women (p<0.05) but not men, likely due to the small sample size. A large number of studies show an inverse association of 25(OH)D and weight in both men and women, thus suggesting that it may be a contributing factor to development or attainment of either an overweight or obese status [7–9]. Plausible explanations as to how this could occur include; inadequate 25(OH)D may be a contributing factor to increasing levels of serum parathyroid (PTH) levels; increased PTH stimulates calcium uptake into fat cells. In turn, this increase in intracellular calcium may stimulate lipogenesis[29]. Excess body fat may act as a storage site for vitamin D, decreasing its bioavailability. Previously, it has been shown that weight loss promotes increased 25(OH)D [30].

Waist circumference is a risk factor for metabolic syndrome and is inversely associated with 25(OH)D [10, 29]. In this group of participants, 31% had higher than desirable waist circumference and were also 25(OH)D insufficient or deficient. These findings are similar to Cheng et al [9], who found that participants in The Framingham Heart Study had an inverse association between 25(OH)D status and visceral adiposity even among lean individuals. Several other investigators have found similar inverse relationships between waist circumference measures and 25(OH)D status [29]. There was a significant association between visceral fat and total fat in this sample (*p*<0.001). Evidence supports that visceral versus subcutaneous adiposity is more detrimental to overall health. It is purported that the free fatty acids produced from the visceral adipocyte have portal drainage into the liver, playing a significant role in the pathogenesis of the metabolic syndrome and that this type of adipose tissue is more likely to produce inflammatory adipokines that are linked to insulin resistance [11].

Concerning intake of our nutrients of interest, we found that 16% had adequate intakes of vitamin D and only 5% with adequate calcium intake. Of the 16% with adequate intake of vitamin D all had normal 25(OH)D except one, who verbalized they had just started taking supplemental vitamin D. Since few foods are rich sources of vitamin D and many individuals do not experience the necessary quantity of sun exposure, routine intake of vitamin D fortified foods and supplements may be necessary to ensure adequate intakes with subsequent normalization of serum 25(OH)D [1, 14, 15]. The 2015 Dietary Guidelines recommends daily intake of vitamin D of 600 IU for adults up to the age of 71 and then 800 IU daily for adults 71 years of age and older to maintain adequate levels of 25(OH)D. Calcium intake is recommended at 1,000 mg per day for men and women up to 50 years of age, and then for women 51 years old and up 1,200 mg per day either through food sources or supplements [14]. Recommendations for those individuals found to be insufficient or deficient in 25(OH)D are suggested as 6,000 IU of vitamin D_2_ or D_3_ daily until the blood levels of 25(OH)D are normalized followed by 1,500–2,000 IU per day regimen for maintenance purposes. The recommendations for obese individuals are even higher at up to 10,000 IU per day until normalization occurs followed by 3,000 to 6,000 IU per day for maintenance [1].

There are several limitations to this study. The first is that this was a descriptive, cross-sectional study with a small sample size of mostly non-Hispanic white participants, limiting the generalizability to the general U.S. population. The small sample size may have limited our ability to find associations between 25(OH)D status and measured variables. Our strength measure was limited to the hand grip assessment. Though there is evidence that this can be indicative of an individual’s overall strength, more robust strength measures such as a 1-repetition max for the bench press and leg press would give a comprehensive assessment that could be compared to other studies [16, 26, 27]. Using a 24-hour dietary recall may not have given a true, routine estimate of chronic intake. As participants prepare to have assessments completed, they may have changed their routine food intake; however, the use of the vitamin D and calcium screener enhanced our ability to assess intake of these two specific nutrients. Lastly, we did not measure routine UVB exposure in these clients which is a known contributor to 25(OH)D [12].

## Conclusions

The high rate of 25(OH)D insufficiency and deficiency in this small sample of adults is of concern. Given the important roles of 25(OH)D in the body, it can be recommended that adults have their serum 25(OH)D assessed at routine physical examinations, to determine if treatment is necessary. This group of participants did not consume adequate amounts of calcium and vitamin D indicating the necessity of nutrition education. Consideration should be given, when possible, to utilizing FMI to categorize adiposity and making subsequent recommendations for weight maintenance or loss. Treatment recommendations for those shown to be insufficient and deficient would include weight loss if necessary along with vitamin D_2_ or D_3_ supplementation in the amount of 6,000–10,000 IU per day [1, 8, 30]. Normalizing serum 25(OH)D through food and supplements has the potential to positively impact several parameters of an individual’s health including weight status, visceral adiposity and waist circumference, strength, and bone health.

## Supporting information

S1 File Supporting information. (XLSX)
